# Improvement of the Working Environment and Daily Work-Related Tasks of Dental Hygienists Working in Private Dental Offices from the Japan Dental Hygienists’ Association Survey 2019

**DOI:** 10.3390/dj9020022

**Published:** 2021-02-19

**Authors:** Yoshiaki Nomura, Yuki Ohara, Yuko Yamamoto, Ayako Okada, Noriyasu Hosoya, Nobuhiro Hanada, Noriko Takei

**Affiliations:** 1 Department of Translational Research, Tsurumi University School of Dental Medicine, Yokohama 230-8501, Japan; hanada-n@tsurumi-u.ac.jp; 2 Japan Dental Hygienists’ Association, Tokyo 169-0071, Japan; yohara@tmig.or.jp (Y.O.); nori@pm-ms.tepm.jp (N.T.); 3 Tokyo Metropolitan Institute of Gerontology, Tokyo 173-0015, Japan; 4 Department of Endodontology, Tsurumi University School of Dental Medicine, Yokohama 230-8501, Japan; yamamoto-y@tsurumi-u.ac.jp (Y.Y.); hosoya-n@tsurumi-u.ac.jp (N.H.); 5 Department of Operative Dentistry, Tsurumi University School of Dental Medicine, Yokohama 230-8501, Japan; okada-a@tsurumi-u.ac.jp

**Keywords:** dental hygienists, job satisfaction, work assignments, workplace environment, Japan

## Abstract

A dental hygienist performs various daily work-related tasks. The aim of this study was to elucidate the daily work-related tasks of Japanese dental hygienists and construct groups to understand the relationships between daily work-related tasks, the attractiveness of dental hygienist work, and the improvement of the working environment. The Japan Dental Hygienists’ Association has conducted a postal survey on the employment status of dental hygienists in Japan every five years since 1981. The data on the implementation of 74 daily work-related tasks in dental offices were analyzed from the survey carried out in 2019. The questionnaires were distributed to 16,722 dental hygienists and 8932 were returned (collection rate: 53.4%). The 3796 dental hygienists working at dental clinics were clearly classified into nine groups. Full-time workers requested a reduced workload. Part-time workers requested better treatment rather than reducing the workload. Salary and human relationships were common problems with the working environment. Full-time workers felt that job security was an attractive feature of the dental hygienist role. The data presented in this study may help with the improvement of working conditions for dental hygienists.

## 1. Introduction

Dental hygienists play an important role in promoting oral health [1,2,3,4,5,6,7,8]. The demand has increased for dental hygienists. Even though the laws of each country regulate the range of work completed by dental hygienists, various skills are required for daily work-related tasks. The demand for dental hygienists has expanded in Japan. For example, the evaluation and improvement of oral health has been introduced in the Japanese national insurance system [9]. Dental hygienists need to have a medical team approach for the improvement of oral hygiene of compromised patients [10,11,12,13,14].

The Japanese law on dental hygienists declares that their main task is the improvement of oral health nationwide. The scope of the work of dental hygienists is oral care and oral health guidance under the supervision of a dentist, cleaning teeth, the mechanical removal of deposits on the tooth surface over the gingival margin, and assistance with dental treatment. Their assistance with dental treatment has been expanded. Therefore, there is a need to understand the situation of dental hygienists. This information is useful to promote a medical team approach for the medical and dental treatment of patients with compromised health. It also available for utilizing limited social capital and influencing policymaking. This information is available for many countries other than Japan. However, gathering information on the real work-related tasks of dental hygienists is not enough.

The Japan Dental Hygienists’ Association conducts a survey of dental hygienists every five years, assessing a wide range of items [15]. In 2019, for dental hygienists working in dental clinics, 74 kinds of daily work-related tasks and requests for an improved working environment were included in the questionnaire. Through these items, the daily working tasks of dental hygienists were elucidated. In addition to descriptive statistics, summarizing the information by statistical modeling is effective for understanding the various daily work-related tasks of dental hygienists.

The aim of this study was to elucidate the daily work-related tasks of Japanese’s dental hygienists and present the information effectively by clustering. There has been no report that has analyzed almost all of the daily work-related tasks of dental hygienists. In addition, requests for improvement of the working environment and the attractiveness of dental hygienists’ work were analyzed. The results of this study may be useful for the improvement of working conditions of dental hygienists and could have an effect on the policymaking process.

## 2. Materials and Methods 

### 2.1. Survey Method

The Japan Dental Hygienists’ Association has conducted a postal survey on the employment status of dental hygienists in Japan every five years since 1981 [15]. As this survey was supported by the Japanese government, it conformed to the national survey guidelines. On 1 October 2019, the questionnaire, including a stamped envelope for return, was distributed to all 16,722 members of the Japan Dental Hygienists’ Association by mail. The return date was set for November 11. 

### 2.2. Questionnaire

The questionnaire used in this study consisted of 104 major items related to demographic factors, employment status, daily work-related tasks, willingness to work, etc. The questionnaire consisted of 40 common major items for all dental hygienists, including requests for the improvement of working conditions and the attractiveness of work of dental hygienists, and 11 major items for the dental hygienists working at dental clinics, including implementation of 74 daily work-related tasks in dental offices. In this study, data on dental hygienists working in dental clinics were analyzed. A total of 74 daily work-related tasks were classified into 3 major categories: preventive dental treatment, assistance work, and dental office management. Preventive dental treatment consisted of 3 items and dental office management consisted of 7 items. Assistance work was subcategorized into 10 categories: medical interviews (5), examinations (11), periodontal treatment (4), oral function (2), restorative procedures (9), orthodontic treatment (7), dental implants (5), medical treatment (5), special care dentistry (9), and health instructions (7). Numbers indicated the items for daily work-related tasks. These tasks are listed with the descriptive statistics in Table S1.

The attractiveness of dental hygienists’ work consisted of 7 items: national license, highly specialized occupation, stable employment, stable income, contribution to people and society, maintaining health and life, and directly helping people. Requests for the improvement of working conditions consisted of 13 items: a rise in salary, reducing workload, working relationships, reduced working hours, flexibility in terms of days off and vacation, improvement of parenting support, improvement of nursing care support, valuing of professionalism, opportunities for improving skills, flexibility in terms of work and working hours, improving medical safety systems, ensuring employment stability, and improvement of the employee benefits system. 

### 2.3. Statistical Analysis

#### 2.3.1. IRT Analysis

A three-parameter logistic model with item response theory (IRT) analysis was applied to calculate the item discriminations, item difficulties, and item guesses for the work-related tasks [15]. Item response and item information curves were presented for each work-related task. The analyses were carried out in R ver. 3.50 software with the LTR and irtoys packages using the following formula:

$$P_{i}\left(\theta \right)=\frac{\left(1-c_{i}\right)}{1+e^{-Da_{i}\left(\theta -b_{i}\right)}}$$

#### 2.3.2. Ordination Analysis

For the classification of daily work-related tasks, we applied t-distributed stochastic neighbor embedding (tSNE) analysis. Scatter plots of each dental hygienist were illustrated by 2 dimensions, obtained by tSNE analysis. Using this scatter plot, classification was carried out and the clusters were named groups. To find the rules of classification, we carried out decision analyses by Quick, Unbiased and Efficient Statistical Tree (QUEST). tSNE analysis was performed by R software ver. 3.50 with vegan, and Rtsne package. Decision analysis was carried out by SPSS v. 24.0 (IBM, Tokyo, Japan). 

#### 2.3.3. Correspondence Analysis

A dataset of cross-tabulation for the prevalence of dental caries was used for network plot and correspondence analysis. Network plot was performed by SPSS Modeler Ver.18.0 (IBM, Tokyo, Japan), and correspondence analysis was performed by SPSS Statistics Ver 24.0 (IBM, Tokyo, Japan).

### 2.4. Ethical Approval and Consent to Participate

This study was approved by the Ethics Committee of the Tsurumi University School of Dental Medicine (approval number: 1837) and conducted in accordance with the Declaration of Helsinki. Informed written consent was obtained from all participants.

## 3. Results

### 3.1. Characteristic of the Subjects Analyzed in This Study

From 8932 dental hygienists, responses were returned (collection rate: 8932/16,722, 53.4%). Their working places were dental clinic—3796 (42.5%), hospital—1307 (14.6%), government—913 (10.2%), nursing home—367 (4.1%), leaving jobs—1063 (11.9%), and other—1486 (16.6%). Their educational backgrounds were graduated from university—6811 (76.3%), master’s course—108 (1.2%), doctoral course—59 (0.7%), junior college—1245 (14.0%), dental hygienist school—6881 (77.1%), and no answer—83 (0.9%). Among the 3796 dental hygienists working at dental clinics, the mean age and career length were 45.3 ± 12.2 years old, median: 47.0 (25th‒75th: 37‒54), and 19.2 ± 11.3 years, median: 20 (25th‒75th: 10‒27), respectively. Age and carrier were not normally distributed by Kolmogorov–Smirnov test. Among them, the number of full-time workers was 2064 (54.4%) and the number of part-time workers was 1732 (45.6%). Descriptive statistics of the items analyzed in this study are presented in Tables S1 and S2.

### 3.2. Analysis of Daily Tasks by Item Response Theory

**Figure 1:**
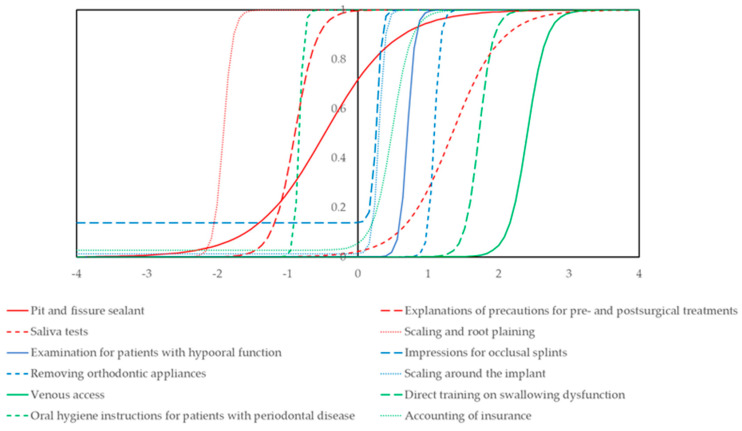
Item response curve of representative dental hygienists’ daily tasks. From all dental hygienists’ daily work-related tasks, the items that had the highest item information in each category are presented. All of the item response and information curves of the daily work-related tasks are shown in Figure S1.

The daily work-related tasks of dental hygienists were analyzed independently by item response theory by the categories presented in Section 2.2. The item response curves that had the highest item information of each category are shown in Figure 1. The item response and item information curves of prevention and assistant works are shown in Figure S1. By the item response theory, we calculated the abilities of the dental hygienists and used them for the following analysis. Models by IRT are shown in Table S3. 

### 3.3. Classification of Daily Tasks by the SNE Analysis

**Figure 2:**
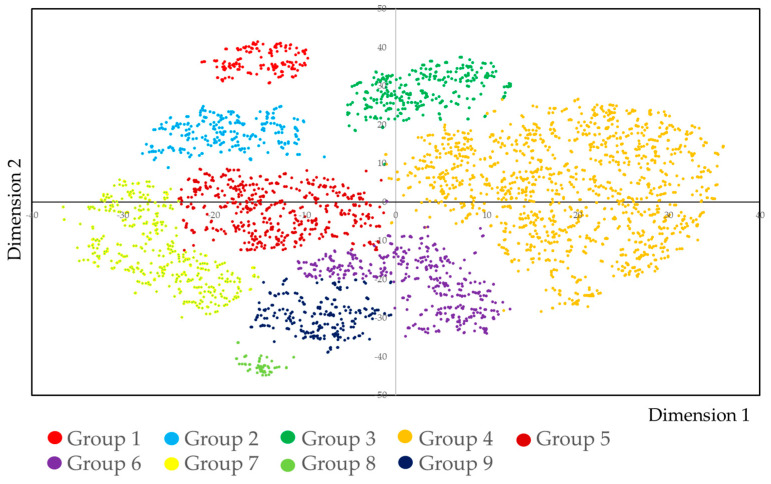
t-distributed stochastic neighbor embedding (tSNE) plot by ability, calculated by item response theory (IRT) analysis of the daily work-related tasks of dental hygienists. Ability indicates the weighted number of implemented daily work-related tasks. Dental hygienists participating in this study were clearly classified by the implementation of daily work-related tasks. tSNE: t-distributed stochastic neighbor embedding.

By using the ability of each job category calculated by IRT analysis, we classified the dental hygienists. For the classification, t-distributed stochastic neighbor embedding (tSNE) analysis was applied. The results are shown in Figure 2. Each dental hygienist was clearly classified by the tSNE analysis. Cross-tabulation of groups and 74 kinds of daily work-related tasks are shown in Table S4. For each group, the mean and median of the ability calculated by IRT analysis are shown in Table S3. In this table, each group is characterized (Table 1). Additionally, the rules of each group were analyzed by Quick, Unbiased and Efficient Statistical Tree (QUEST) analysis. The results are shown in Figure S2 and were consistent with the results shown in Table 1. 

The characteristic daily work-related tasks implemented in each group were as follows:

Group 1: Medical treatment, special care dentistry, and dental implants;
Group 2: Orthodontic treatment, preventive dental care, and periodontal treatment;
Group 3: Dental implants, orthodontic treatment, preventive dental care, and periodontal treatment;
Group 4: Health instructions, preventive dental care, and periodontal treatment;
Group 5: Preventive dental care and periodontal treatment;
Group 6: Preventive dental care;
Group 7: Periodontal treatment;
Group 8: Specialized orthodontic treatment;
Group 9: No specific tasks.

Cross-tabulation of the group and employment status is shown in Table S5. The proportion of part-time workers was higher in groups 4, 5, 6, 7, and 9.

### 3.4. The Contribution of Daily Tasks to the Attractiveness of Dental Hygienists’ Work, and Improvement of Working Environment

#### 3.4.1. Contribution of Daily Tasks to the Improvement of the Working Environment

**Figure 3:**
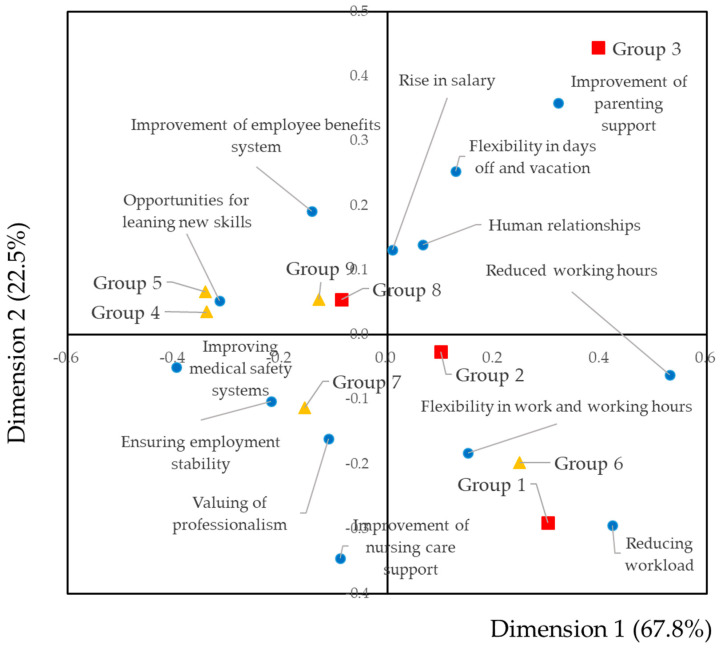
Biplot of groups by daily tasks and requests for improvement of the working environment. The results of the corresponding analysis are graphically illustrated as a biplot. Items with similar characteristics are plotted closely and the smaller the angle in relation to a given point, the stronger the relation. Blue circles indicate requested improvements of the working conditions. Red squares indicate groups in which the proportion of full-time workers is high. Yellow triangles indicate groups in which the proportion of part-time workers is high. The contribution ratio of dimension 1 was 67.8% and for dimension 2 it was 22.5%.

The questionnaire asked about requested improvements of the working environment and the attractiveness of dental hygienists’ work, with 14 and 7 items, respectively. For these items, cross-tabulations were constructed against the groups. The results are shown in Table S5. To visualize the results, we carried out a correspondence analysis. The results of requested improvements to the working environment are shown in Figure 3.

Groups 1 and 6 were surrounded by reduced working hours, reduced workload, and flexibility of work and working hours. The angle between flexibility of work in groups 1 and 6 were smaller than in group 2. This indicated that the correlations flexibility of work in groups 1 and 6 were stronger than group 2. Group 1, in which the proportion of full-time workers was high, was located near the reduced working load. The opportunity to improve one’s skills was located near groups 4 and 5, in which the proportion of part-time workers was high. Ensuring employment stability and a rise in salary were located near groups 8 and 9. The correlations between ensuring employment stability in group 7 was stronger than in groups 8 and 9.

#### 3.4.2. Contribution of Daily Tasks to the Attractiveness of Dental Hygienists’ Work

**Figure 4:**
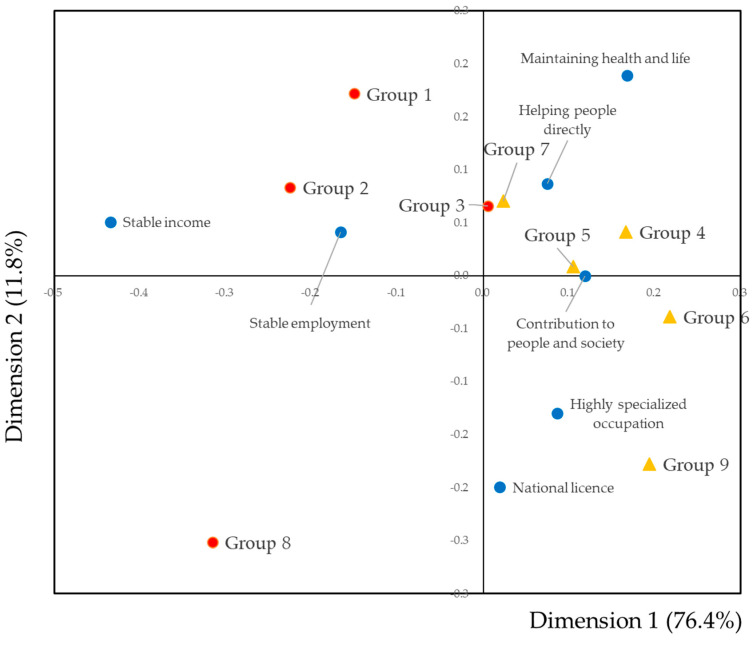
Biplot of groups by daily tasks and the attractiveness of dental hygienists’ work. Blue circles indicate the attractiveness of dental hygienists’ work. Red squares indicate groups in which the proportion of full-time workers were high. Yellow triangles indicate groups in which the proportion of part-time workers were high. The contribution ratio of dimension 1 was 76.4% and that of dimension 2 was 11.8%.

A biplot of the groups and the attractiveness of dental hygienists’ work is shown in Figure 4. Stable income and stable employment were in the negative direction. When focused on dimension 1, groups 1, 2, and 8, in which the proportion of full-time workers was high, were located in the negative direction. Associations between stable income and employment with these groups were higher than other groups. These results indicated that the values of the regular employee were different from part-time workers.

## 4. Discussion

In this study, 74 of Japanese dental hygienists’ daily work-related tasks were analyzed by statistical modeling. Dental hygienists were clustered by the implementation of these tasks.

The role of a dental hygienist covers both clinical and health promotion responsibilities. The clinical practices allowed by law vary between countries. In the USA, the work performed by dental hygienists differs between states. Most states allow scaling, fluoride application, and pit and fissure sealants. Some states allow X-ray exposure. In the UK, scaling, tooth cleaning, pit and fissure sealants, impressions, X-ray exposure, and administering a local anesthetic are allowed. In Japan, fit and fissure sealants, X-ray exposure, impressions, and administering a local anesthetic are not allowed. Only assisting a dentist with these procedures is allowed. Therefore, in order to extrapolate the results of this study to countries other than Japan, one may refer to Figure S1 as a useful tool. All of the tasks, including assisting dentists, were analyzed by IRT.

Professional and personal life, income and job security, and quality of service are all important factors affecting the job satisfaction of dental hygienists [16]. The characteristics of the groups in terms of the attractiveness of the job and requests for improvements of the working environment were investigated. The item response and item information curves presented in Figure S1 provide valuable information beyond simple descriptive statistics.

Item information curves located in the negative direction and with high item information were “prophylactic calculus removal” (Figure S1A), “scaling and root planing” (Figure S1D), and “oral hygiene instruction for patients with periodontal disease” (Figure S1K). The frequencies of these tasks were 3593 (94.7%), 3681 (97.0%), and 3258 (92.8%), respectively. These results indicated that most dental hygienists implemented these tasks. Initially, dental hygienists performed prophylaxis [17]. If dental hygienists implemented these tasks, they also implemented other tasks within the preventive dental treatment, periodontal treatment, and medical and dental guidance. Consistent with previous study, these tasks were common in the groups presented in Table 1.

In contrast to these tasks, item response and item information curves were located in the forward direction for “examination” (Figure S1C), “orthodontic treatment” (Figure S1G), “medical treatment” (Figure S1I), and “special care dentistry” (Figure S1J). These tasks were implemented by a limited number of dental hygienists. When dental hygienists implemented the tasks with high item information, they implemented other tasks within the groups of tasks.

In addition to these tasks, dental hygienists implemented dental office management. “Sterilization and disinfection of dental equipment” was implemented by 3353 (88.3%) of dental hygienists and “management and ordering of drugs and dental equipment” by 2930 (77.1%). These tasks are not necessarily specialties of dental hygienists. Division of labor is necessary to reduce the workload of dental hygienists.

From the tSNE analysis, we found that dental hygienists were clearly classified. For the groups and requests for improvement of working conditions, full-time workers were located in the forward direction and part-time workers were located in the negative direction when we focused on dimension 1. Reducing working hours and reducing workload were located in the forward direction. The results indicated that full-time dental hygienists work too hard and thus reducing the workload is necessary. A previous report had shown that 68% of dental hygienists experience unmanageable workloads [18]. In this study, 1625 (42.9%) dental hygienists answered “Yes” to the question about “reducing workload.” The difference may be derived from the difference in proportion of part-time workers. The part-time workers requested “opportunities for improving one’s skills,” “improvement of employee benefits system,” “ensuring employment stability,” and “valuing professionalism.” Dental hygienists sought to expand their activities [19]. Dental hygienists actively participated in continuing education after career breaks and engaged as part-time workers [20]. These results suggest that, for part-time workers, better treatment was more important than reducing their workload. It was related to the job satisfaction of dental hygienists [18]. Dental hygienists’ workplace satisfaction was associated with their salary [18,20]. A rise in salary was located in the center of dimension 1. This indicates that getting a raise is a common problem, independent of the groups classified by the tSNE analysis. Psychosocial health is important for dental hygienists [21,22]. Human relationships were located near the center, indicating that they represent a common problem for dental hygienists [23]. The results were consistent with previous studies.

In terms of the attractiveness of dental hygienists’ work and groups, full-time workers were located in the negative direction and part-time workers in the forward direction. “Stable income” and “stable employment” were located in the negative direction. Full-time workers felt that job security was an attractive feature of dental hygienists’ work. In contrast, “maintaining health and life,” “contribution to people and society,” and “helping people directly” were located in the forward direction. Part-time workers felt the work was more attractive than full-time workers.

There are several limitations of this study. The response rate was not high, and the sample only included members of the Japan Dental Hygienists’ Association. The members of the Japan Dental Hygienists’ Association are dental hygienists who are willing to study and improve their skills. This included a small number of dental hygienists who had lost their willingness to work. However, this may be the first report to analyze the daily work-related tasks of dental hygienists and their association with job satisfaction. The work-related tasks of dental hygienists vary between countries. The results of each task are applicable to countries other than Japan.

## 5. Conclusions

The attractiveness of dental hygienists’ work and requests for improvement of the working environment largely depend on the daily work-related tasks and working style. To improve the job satisfaction of dental hygienists, there must be an improvement in working conditions corresponding to the daily work-related tasks and working style. The groups constructed by daily work-related tasks may help with the effective improvement of working conditions of dental hygienists.

## Figures and Tables

**Table 1:** Characteristics of the groups by tSNE analysis.

		Group 1	Group 2	Group 3	Group 4	Group 5	Group 6	Group 7	Group 8	Group 9
		*n* = 156	*n* = 264	*n* = 299	*n* = 1381	*n* = 529	*n* = 449	*n* = 428	*n* = 55	*n* = 235
Preventive dental care		+	+	+	+	+	+			
Assistance work	Periodontal treatment	+	+	+	+	+		+		
	Medical treatment	+								
	Special care dentistry	+								
	Orthodontic treatment		+	+					+	
	Dental implants	+		+						
	Health instructions	+	+	+	+					

The “+” show that the median of ability exceeded the 75th percentile of ability calculated for the whole sample. This indicates that dental hygienists in each group implemented the daily work-related tasks more frequently than the third quartile. Group 9 had no characteristic tasks. Median and mean values of all daily work-related tasks are shown in Table S3.

## Data Availability

Data are available from the corresponding author upon reasonable request.

## Supplementary Materials

The following are available online at https://www.mdpi.com/2304-6767/9/2/22/s1, Figure S1: Item response and item information curves of daily tasks of dental hygienists. Figure S2: Decision tree to find out the groups classified by tSNE analysis. Table S1: Cross-tabulation of groups and 74 dental hygienists’ daily work-related tasks in dental offices. Table S2: Cross-tabulation of items related to the attractiveness of dental hygienists’ work, and the improvement of working conditions. Table S3: Results of three parameter logistic models for daily work-related tasks. Table S4: Mean and median of the ability calculated by IRT analysis of each group. Table S5: Employment status in each group.
