# Supplementary material for: Improvement of the Working Environment and Daily Work-Related Tasks of Dental Hygienists Working in Private Dental Offices from the Japan Dental Hygienists’ Association Survey 2019
Source: Dent J (Basel). 2021 Feb 19;9(2):22. doi: 10.3390/dj9020022 (PMC7923276; doi:10.3390/dj9020022)
Supplement: Supplementary file 1 [file dentistry-09-00022-s001.pdf]

Supplemental material

(A)

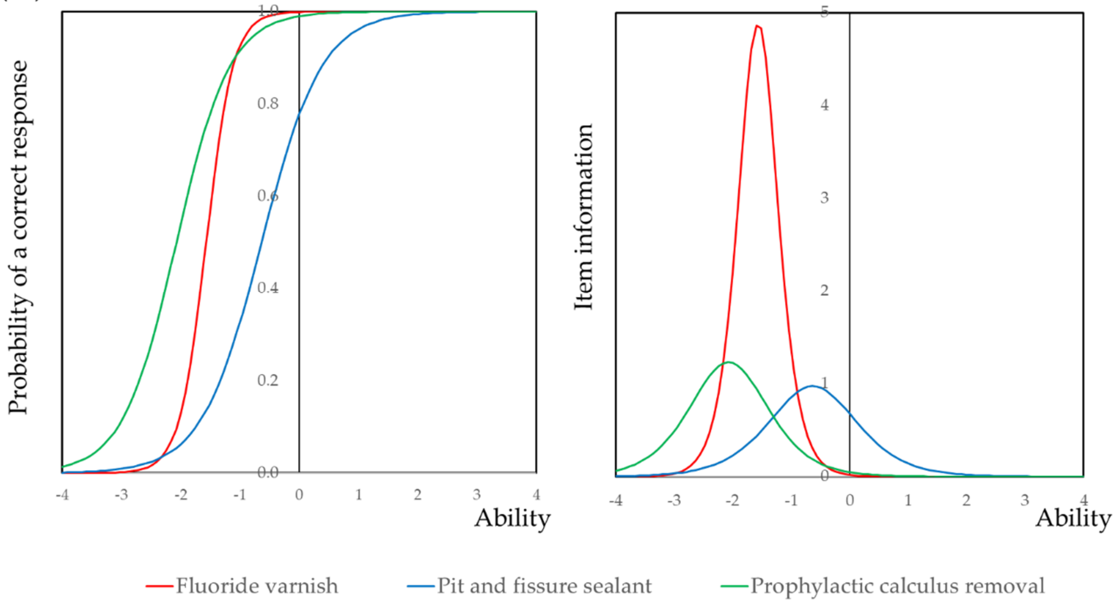

(B)

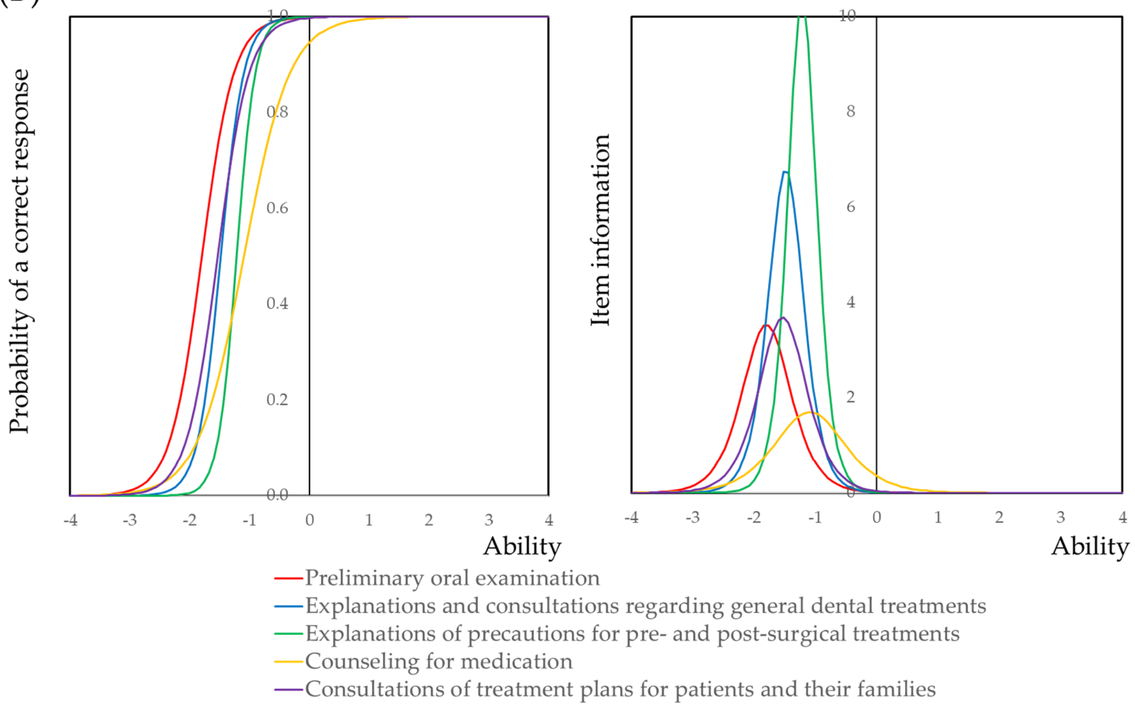

(C)

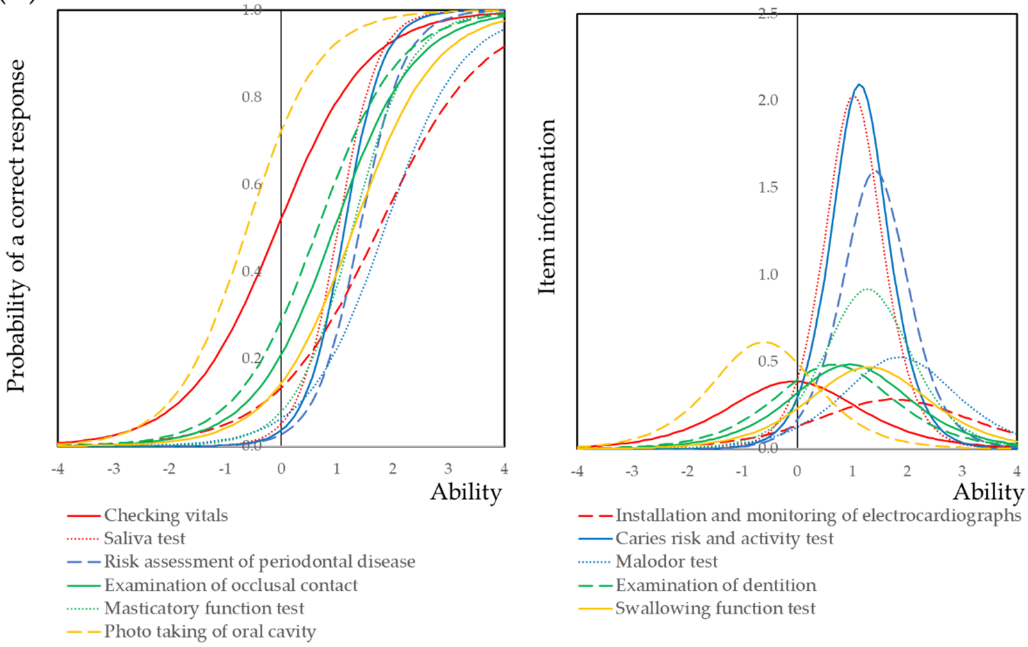

Probability of a correct response
1.0
0.8
0.6
0.4
0.2
0.0
-4
-3
-2
-1
0
1
2
3
4
Ability
Item information
2.5
2.0
1.5
1.0
0.5
0.0
Ability
Checking vitals
Saliva test
Risk assessment of periodontal disease
Examination of occlusal contact
Masticatory function test
Photo taking of oral cavity
Installation and monitoring of electrocardiographs
Caries risk and activity test
Malodor test
Examination of dentition
Swallowing function test

(D)

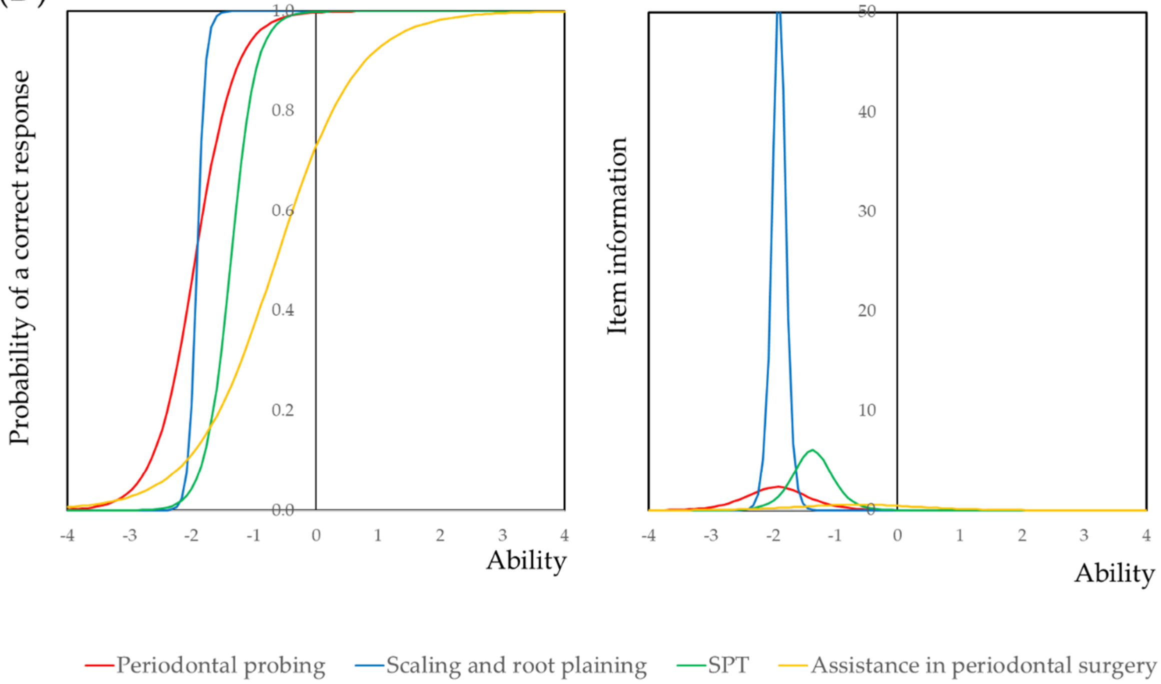

Probability of a correct response
1.0
0.8
0.6
0.4
0.2
0.0
-4
-3
-2
-1
0
1
2
3
4
Ability
Item information
50
40
30
20
10
0
Ability
Periodontal probing
Scaling and root plaining
SPT
Assistance in periodontal surgery

(E)

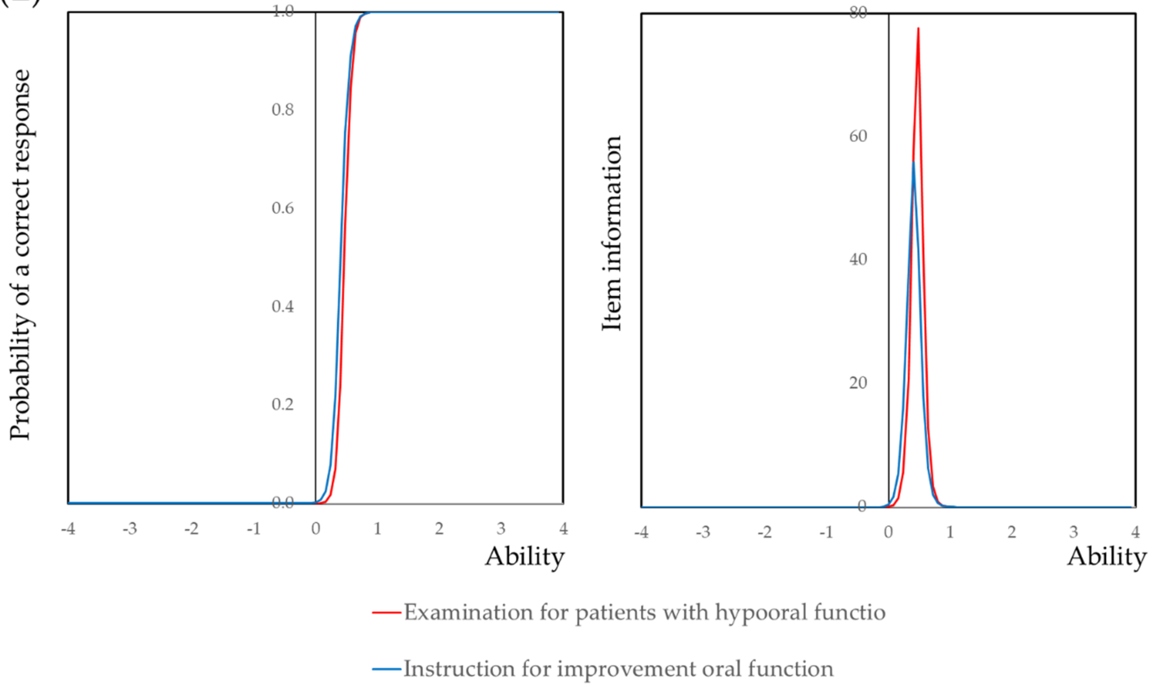
Probability of a correct response
1.0
0.8
0.6
0.4
0.2
0.0
-4
-3
-2
-1
0
1
2
3
4
Ability
Item information
80
60
40
20
0
Ability
Examination for patients with hypooral functio
Instruction for improvement oral function

(F)

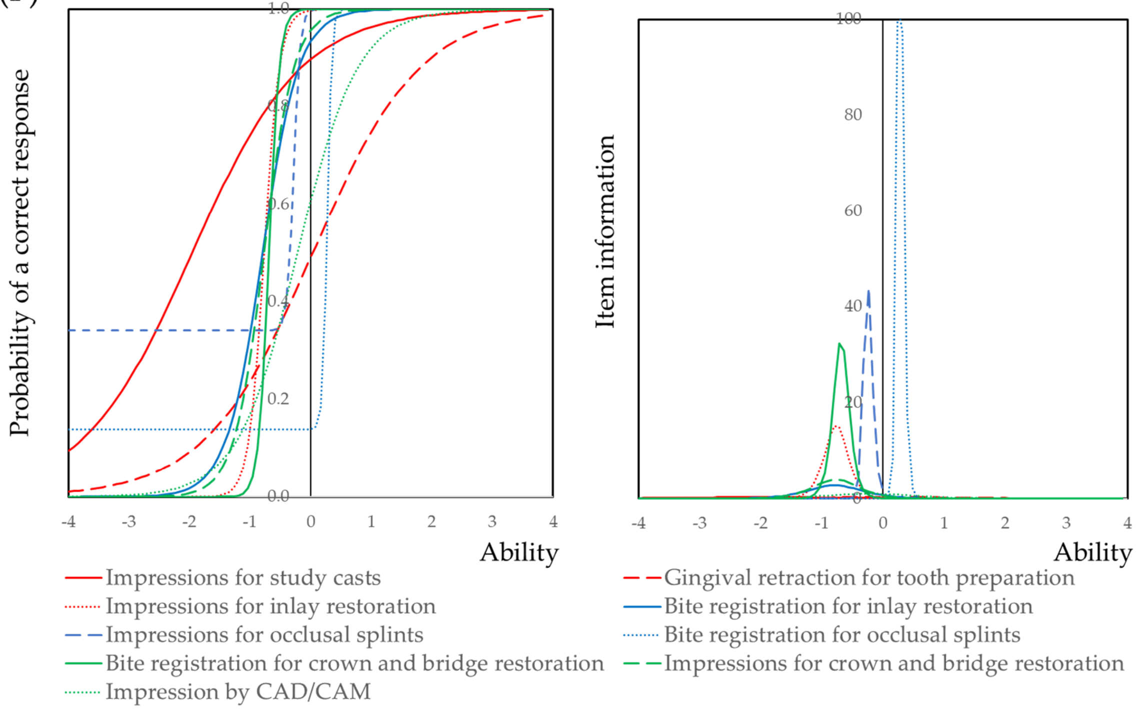
Probability of a correct response
1.0
0.8
0.6
0.4
0.2
0.0
-4
-3
-2
-1
0
1
2
3
4
Ability
Item information
100
80
60
40
20
0
Ability
Impressions for study casts
Gingival retraction for tooth preparation
Impressions for inlay restoration
Bite registration for inlay restoration
Impressions for occlusal splints
Bite registration for occlusal splints
Bite registration for crown and bridge restoration
Impressions for crown and bridge restoration
Impression by CAD/CAM

(G)

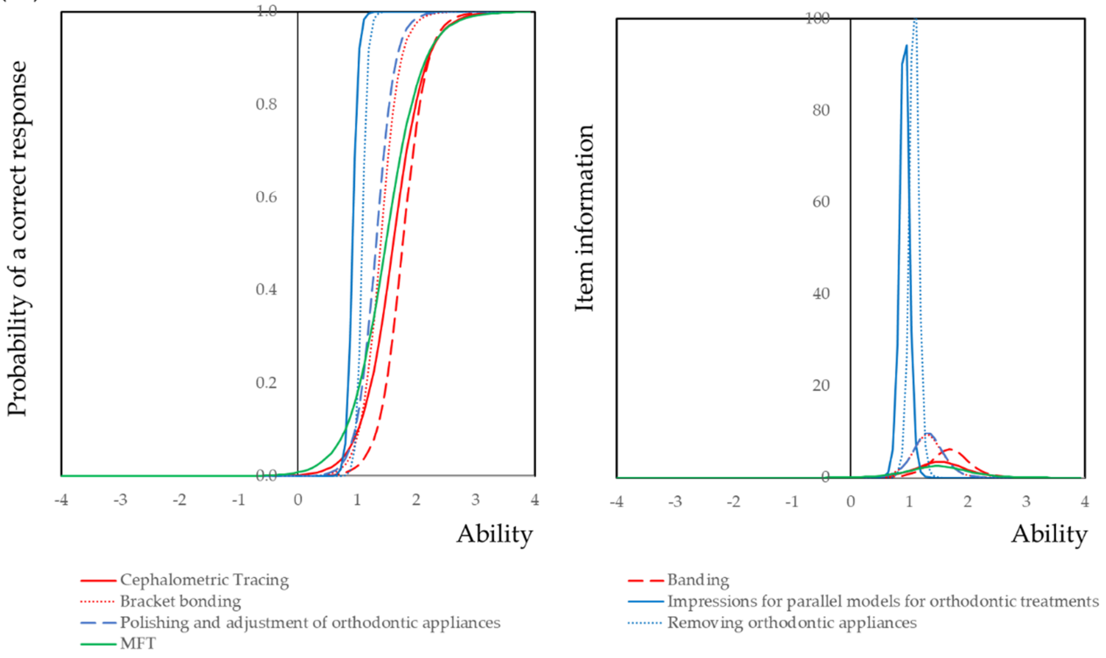

Probability of a correct response
Ability
Item information
Ability
Cephalometric Tracing
Bracket bonding
Polishing and adjustment of orthodontic appliances
MFT
Banding
Impressions for parallel models for orthodontic treatments
Removing orthodontic appliances

(H)

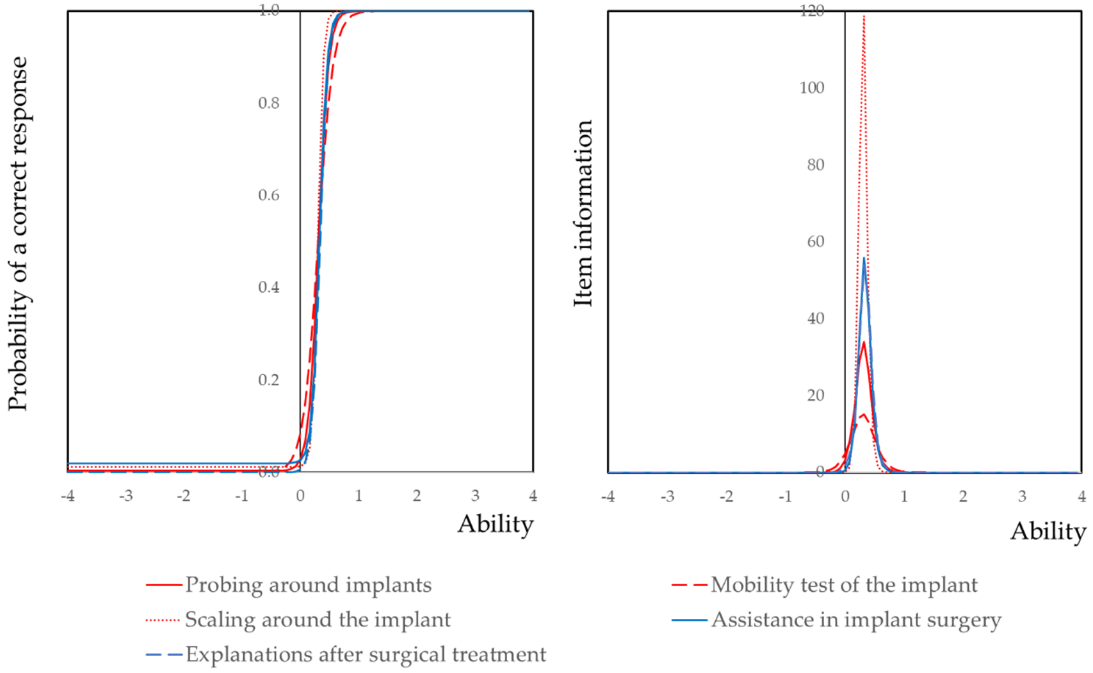

Probability of a correct response
Ability
Item information
Ability
Probing around implants
Scaling around the implant
Explanations after surgical treatment
Mobility test of the implant
Assistance in implant surgery

(I)

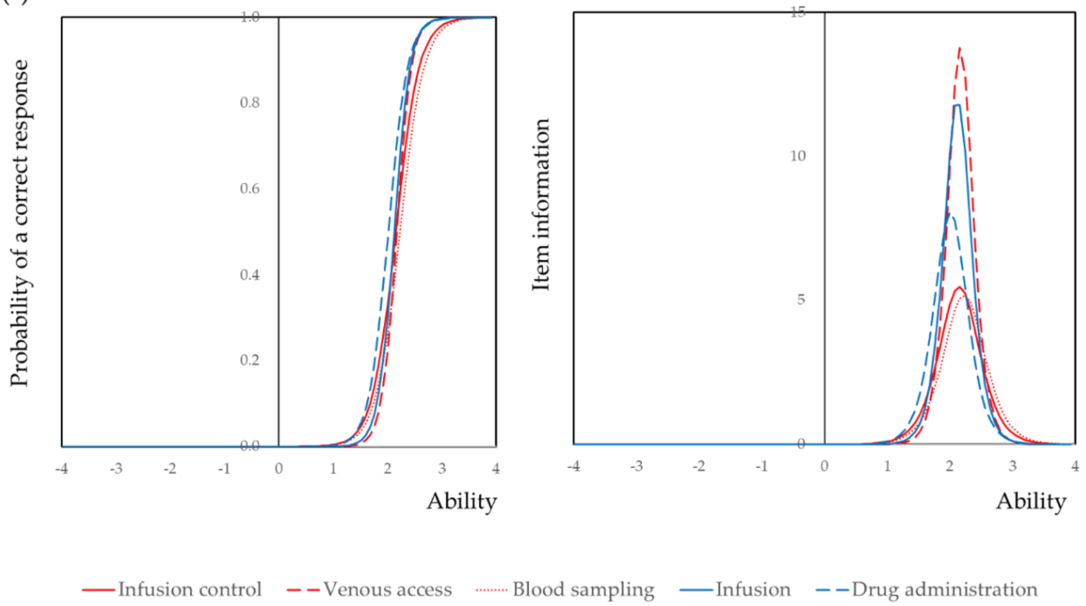

Probability of a correct response
1.0
0.8
0.6
0.4
0.2
0.0
-4
-3
-2
-1
0
1
2
3
4
Ability
Item information
15
10
5
0
Ability
Infusion control
Venous access
Blood sampling
Infusion
Drug administration

(J)

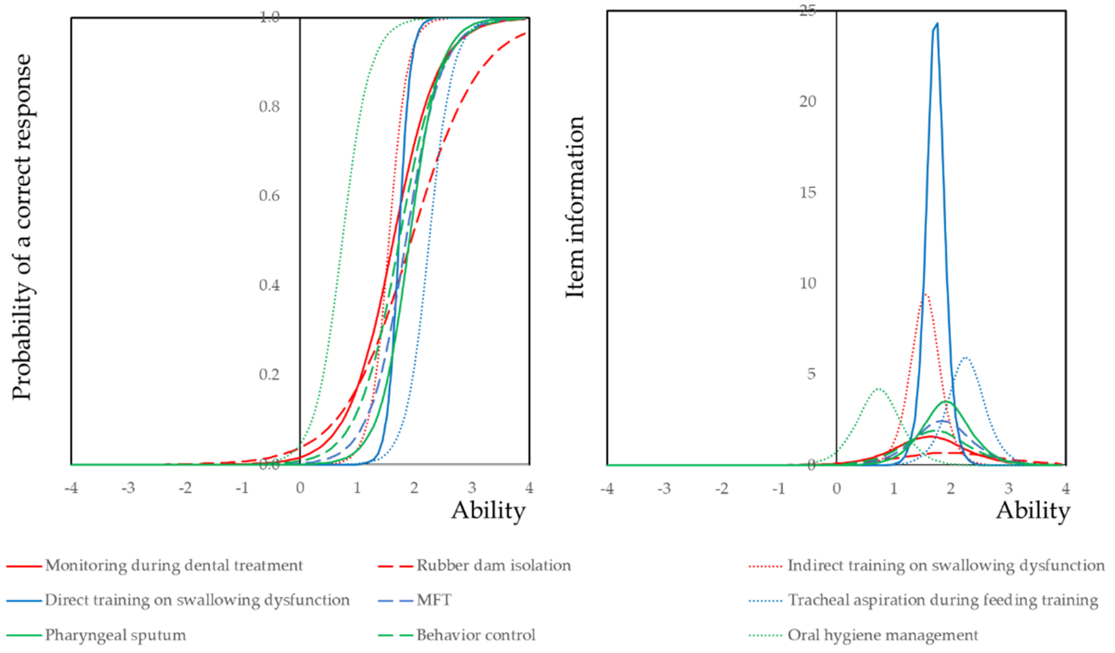

Probability of a correct response
1.0
0.8
0.6
0.4
0.2
0.0
-4
-3
-2
-1
0
1
2
3
4
Ability
Item information
25
20
15
10
5
0
Ability
Monitoring during dental treatment
Rubber dam isolation
Indirect training on swallowing dysfunction
Direct training on swallowing dysfunction
MFT
Tracheal aspiration during feeding training
Pharyngeal sputum
Behavior control
Oral hygiene management

(K)

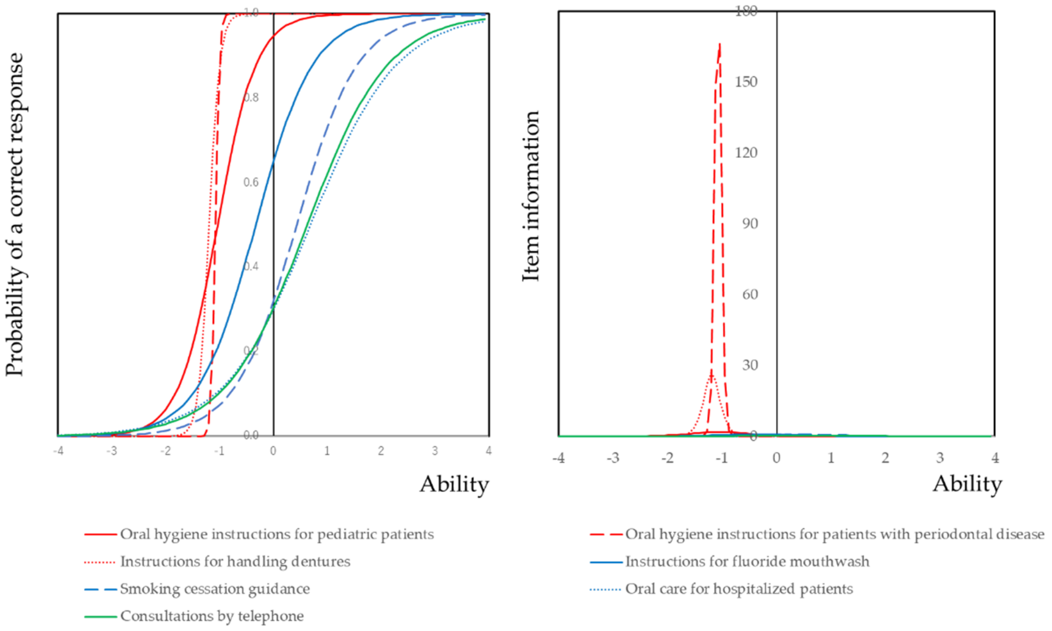

(L)

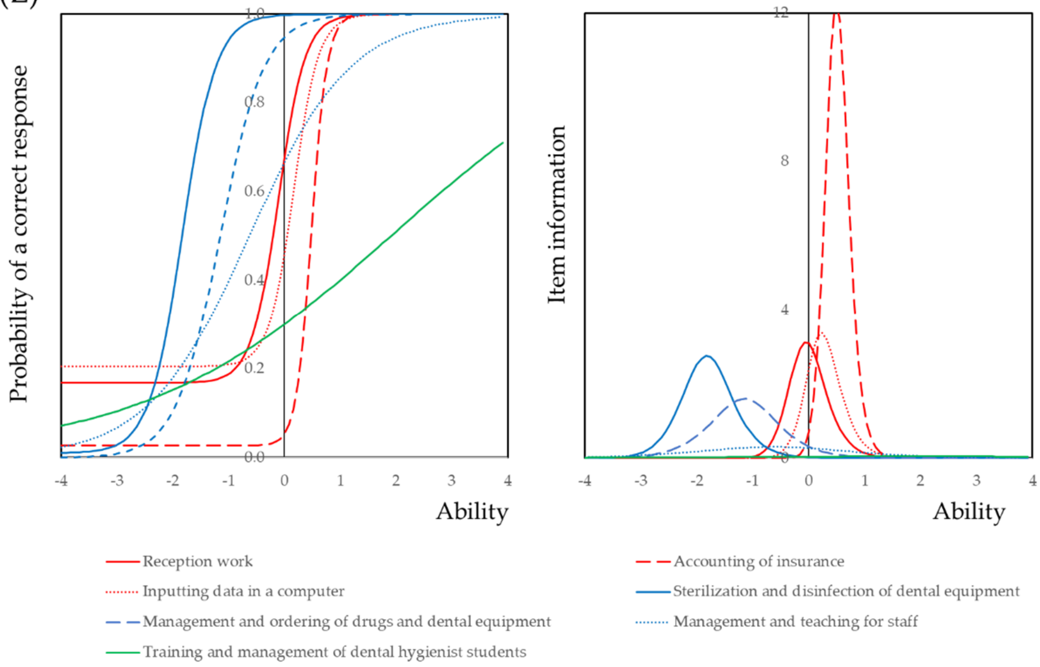

Figure S1. Item response curve and item information curves of daily jobs of dental hygienists.

(A): Oral hygiene treatment, (B): Medical Interview, (C): Examination (D): Periodontal treatment, (E): Improvement of oral function, (F): Restorative procedures, (G): Orthodontic treatment, (H): Dental implant, (I): Medical treatment, (J): Special care dentistry, (K): Medical and dental guidance, (L): Dental office management

Group	n	%
1	156	4.1%
2	264	7.0%
3	299	7.9%
4	1318	36.4%
5	529	13.9%
6	449	11.8%
7	428	11.3%
8	55	1.4%
9	235	6.2%

Ability
(Special care dentistry)

< 0.345

Group	n	%
1	2	0.1%
2	264	7.3%
3	299	8.2%
4	1379	37.9%
5	529	14.5%
6	448	12.3%
7	428	11.8%
8	55	1.5%
9	235	6.3%

> 0.345

Group	n	%
1	154	98.1%
2	0	0.0%
3	0	0.0%
4	2	1.3%
5	0	0.0%
6	1	0.6%
7	0	0.0%
8	2	1.3%
9	0	0.0%

Ability
(Dental implant)

< 0.578

Group	n	%
1	2	0.1%
2	8	0.3%
3	8	0.3%
4	1335	45.2%
5	521	17.6%
6	436	14.8%
7	410	13.9%
8	3	0.1%
9	229	7.8%

> 0.578

Group	n	%
1	0	0.0%
2	256	37.3%
3	291	42.4%
4	44	6.4%
5	8	1.2%
6	12	1.7%
7	18	2.60%
8	52	7.6%
9	6	0.9%

Ability
(Preventive dental treatment)

< -1.250

Group	n	%
1	0	0.0%
2	0	0.0%
3	0	0.0%
4	129	43.7%
5	0	0%
6	59	20.0%
7	53	18.0%
8	0	0%
9	54	18.3%

> -1.250

Group	n	%
1	2	0.1%
2	8	0.3%
3	8	0.3%
4	1026	45.4%
5	521	19.6%
6	377	14.2%
7	357	13.4%
8	3	0.1%
9	175	6.6%

Ability
(Orthodontics)

< -0.838

Group	n	%
1	0	0.0%
2	20	20.4%
3	11	11.2%
4	10	10.2%
5	2	2.0%
6	4	4.1%
7	5	5.1%
8	46	46.9%
9	0	0%

> -0.838

Group	n	%
1	0	0.0%
2	236	40.1%
3	280	47.5%
4	34	5.8%
5	6	1.0%
6	8	1.4%
7	13	2.2%
8	6	1.0%
9	6	1.0%

Ability
(Periodontal treatment)

<-1.43

Group	n	%
1	0	0.0%
2	0	0.0%
3	0	0.0%
4	43	44.8%
5	0	0.0%
6	52	54.2%
7	1	1.0%
8	0	0.0%
9	0	0.0%

> -1.3

Group	n	%
1	0	0.0%
2	0	0.0%
3	0	0.0%
4	86	43.2%
5	0	0%
6	7	3.5%
7	52	26.1%
8	0	0%
9	54	27.1%

Ability
(Preventive dental treatment)

< -0.783

Ability
(Periodontal treatment)

< -0.929

Group	n	%
1	0	0.0%
2	0	0.0%
3	0	0.0%
4	30	33.0%
5	0	0%
6	57	62.6%
7	3	3.3%
8	0	0%
9	1	1.1%

> -0.929

Group	n	%
1	1	0.1%
2	5	0.7%
3	3	0.4%
4	287	42.3%
5	4	0.6%
6	21	3.0%
7	203	29.9%
8	0	0.0%
9	155	22.8%

> -0.783

Group	n	%
1	1	0.1%
2	3	0.2%
3	5	0.3%
4	889	47.1%
5	517	27.4%
6	289	15.8%
7	151	8.0%
8	3	0.2%
9	19	1.0%

Figure S2. Decision tree to find out the Groups classified by tSNE analysis

Table S1. Cross tabulation of Groups and 74 dental hygienist daily work related tasks in dental office

			Group									Total
			1	2	3	4	5	6	7	8	9	n=3796
			n=156	n=264	n=299	n=1381	n=529	n=449	n=428	n=55	n=235	
Preventive dental treatment		Fluoride varnish	152	259	299	1274	529	390	384	41	191	3519
		Pit and fissure sealant	121	206	262	936	523	313	178	17	39	2595
		Prophylactic calculus removal	155	259	296	1292	529	409	400	43	210	3593
Assistant works	Medical Interview	Preliminary oral examination	153	263	297	1302	528	343	424	48	231	3589
		Explanations and consultations regarding general dental treatments	154	262	298	1261	514	295	422	53	226	3485
		Explanations of precautions for pre- and post-surgical treatments	153	261	294	1210	496	255	405	49	228	3351
		Counseling for medication	145	230	271	1150	455	244	361	47	198	3101
		Consultations of treatment plans for patients and their families	155	261	298	1266	504	296	416	54	227	3477
	Examination	Checking vitals	138	151	223	813	239	100	225	12	69	1970
		Installation and monitoring of electrocardiographs	100	52	85	211	87	16	100	2	14	667
		Saliva test	56	67	98	271	112	27	70	17	12	730
		Caries risk and activity test	43	68	87	220	93	20	68	19	11	629
		Risk assessment of periodontal disease	38	40	62	171	69	12	54	3	9	458
		Malodor test	40	46	77	151	48	16	47	1	14	440
		Examination of occlusal contact	68	114	115	368	144	39	138	20	32	1038
		Examination of dentition	79	148	173	423	181	61	146	42	38	1291
		Masticatory function test	50	51	94	297	69	12	55	8	13	649
		Swallowing function test	42	40	108	423	61	25	63	5	21	788
		Photo taking of oral cavity	140	234	277	903	380	151	297	52	81	2515
	Periodontal treatment	Periodontal probing	156	264	297	1299	529	375	428	31	213	3592
		Scaling and root plaining	156	263	299	1329	529	414	428	38	225	3681
		SPT	153	259	295	1221	527	338	427	22	161	3403
		Assistance in periodontal surgery	136	212	241	919	494	86	391	0	37	2516
	Oral function	Examination for patients with hypo oral function	53	48	140	555	86	32	95	7	18	1034
		Instruction for improvement oral function	56	64	154	628	116	30	128	11	32	1219
	Restorative procedures	Impressions for study casts	145	251	287	1143	480	298	394	48	172	3218
		Gingival retraction for tooth preparation	103	150	205	685	266	118	237	1	75	1840
		Impressions for inlay restoration	134	228	271	1040	420	272	335	4	158	2862
		Bite registration for inlay restoration	132	235	276	1017	418	268	326	3	150	2825
		Impressions for occlusal splints	134	224	268	960	404	228	318	25	114	2675
		Bite registration for occlusal splints	109	177	221	754	308	149	249	16	80	2063
		Impressions for crown and bridge restoration	136	226	276	1017	418	266	328	5	155	2827
		Bite registration for crown and bridge restoration	134	233	279	1017	421	266	322	4	153	2829
		Impression by CAD/CAM	114	180	232	755	316	192	229	7	85	2110
	Orthodontic treatment	Cephalometric Tracing	40	99	128	10	1	2	2	29	0	311
		Banding	36	68	88	8	1	0	0	22	0	223
		Bracket bonding	52	112	162	12	1	1	1	27	2	370
		Impressions for parallel models for orthodontic treatments	62	246	289	40	7	9	15	50	6	724
		Polishing and adjustment of orthodontic appliances	50	102	164	14	1	1	2	31	2	367
		Removing orthodontic appliances	51	138	179	17	3	1	3	41	2	435
		MFT	40	107	146	14	3	1	4	37	1	353
	Dental	Probing around implants	116	162	187	462	220	66	209	3	24	1449

	implant	Mobility test of the implant	117	166	186	450	218	63	197	3	23	1423
		Scaling around the implant	115	171	218	505	249	84	225	3	36	1606
		Assistance in implant surgery	127	170	213	440	232	48	199	3	26	1458
		Explanations after surgical treatment	128	164	206	411	218	25	190	3	23	1368
	Medical treatment	Infusion control	82	0	0	0	0	0	0	0	0	82
		Venous access	63	0	0	0	0	0	0	0	0	63
		Blood sampling	70	0	0	1	0	0	0	0	0	71
		Infusion	68	0	0	1	0	1	0	0	0	70
		Drug administration	96	0	0	2	0	0	0	0	0	98
	Special care dentistry	Monitoring during dental treatment	49	15	62	157	24	9	21	4	6	347
		Rubber dam isolation	41	27	67	115	33	11	26	8	12	340
		Indirect training on swallowing dysfunction	22	5	53	168	5	3	10	3	0	269
		Direct training on swallowing dysfunction	18	3	33	121	4	1	4	1	0	185
		MFT	26	10	50	80	9	3	9	11	2	200
		Tracheal aspiration during feeding training	10	0	11	24	1	1	4	0	0	51
		Pharyngeal sputum	18	2	22	96	6	1	7	1	0	153
		Behavior control	32	17	41	120	23	12	18	4	7	274
		Oral hygiene management	76	61	118	453	85	42	72	12	26	945
	Health instructions	Oral hygiene instructions for pediatric patients	136	234	284	1077	451	298	346	30	166	3022
		Oral hygiene instructions for patients with periodontal disease	144	240	290	1210	470	327	375	17	185	3258
		Instructions for handling dentures	148	239	291	1262	475	320	374	6	194	3309
		Instructions for fluoride mouthwash	110	174	240	855	325	155	257	18	91	2225
		Smoking cessation guidance	78	114	158	550	177	73	189	3	66	1408
		Oral care for hospitalized patients	71	55	190	723	71	33	98	0	30	1271
		Consultations by telephone	77	92	153	577	140	54	150	9	47	1299
Dental office management		Reception work	96	152	196	788	294	194	264	36	157	2177
		Accounting of insurance	61	79	128	419	144	67	131	17	73	1119
		Inputting data in a computer	95	144	204	725	266	149	231	27	116	1957
		Sterilization and disinfection of dental equipment	146	236	285	1212	468	343	399	50	214	3353
		Management and ordering of drugs and dental equipment	132	216	268	1062	406	270	354	48	174	2930
		Management and teaching for staff	120	168	235	833	314	148	271	38	111	2238
		Training and management of dental hygienist students	81	84	159	406	119	47	127	20	32	1075

Table S2. Cross tabulation of for items of attractiveness of dental hygienists works, and improvement of working environments

		Group 1	Group 2	Group 3	Group 4	Group 5	Group 6	Group 7	Group 8	Group 9	Total
		n=156	n=264	n=299	n=1,381	n=529	n=449	n=428	n=55	n=235	n=3,796
Attractiveness of dental hygienists works	National license	154	259	289	1329	506	426	417	55	228	3663
	Highly specialized occupation	148	248	279	1322	490	410	400	51	217	3565
	Stable employment and job change	141	224	237	1091	408	332	341	46	173	2993
	Stable income	117	201	199	844	323	250	287	40	134	2395
	Contribution to people and society	150	235	276	1271	479	391	402	48	208	3460
	Maintaining health and life	149	241	275	1278	479	394	397	44	200	3457
	Direct help for people	148	233	261	1239	444	383	380	46	193	3327
Request of improvement of working environments	Rising in salary	114	191	227	1014	394	324	320	43	171	2798
	Reducing workload	87	115	158	603	215	163	176	25	83	1625
	Working relationship	61	109	135	562	215	191	164	24	88	1549
	Reducing working hours	77	101	130	487	194	140	149	25	73	1376
	Flexibility in day off and vacation	74	124	143	636	250	203	216	30	102	1778
	Improvement of parenting support	56	88	104	419	169	136	150	24	71	1217
	Improvement of nursing care support	71	104	127	568	211	186	175	18	87	1547
	Valuation of professionalism	92	145	172	835	300	252	254	27	128	2205
	Opportunities for skill up	76	122	140	731	259	245	225	25	131	1954
	Flexibility in work and working hours	73	113	141	558	208	171	185	22	89	1560
	Improving medical safety systems	79	130	146	787	285	267	241	24	138	2097
	Ensuring employment stability	76	115	146	694	260	235	217	23	113	1879
	Improvement of employee benefits system	90	140	163	814	333	279	272	34	134	2259

Numbers in cells are number of subjects who answered "yes" for the items

Table S3. Results of three parameter logistic model for daily work related tasks

Category		Jobs	Discrimination	Difficulty	Guessing
Preventive dentistry		Fluoride varnish	4.42	-1.57	<0.01
		Pit and fissure sealant	1.97	-0.63	<0.01
		Prophylactic calculus removal	2.22	-2.07	<0.01
Assistant work	Medical Interview	Preliminary oral examination	3.76	-1.80	<0.01
		Explanations and consultations regarding general dental treatments	5.22	-1.48	<0.01
		Explanations of precautions for pre- and post-surgical treatments	6.45	-1.22	<0.01
		Counseling for medication	2.61	-1.09	<0.01
		Consultations of treatment plans for patients and their families	3.84	-1.53	<0.01
	Examination	Checking vitals	1.25	-0.07	<0.01
		Installation and monitoring of electrocardiographs	1.07	1.75	<0.01
		Saliva test	2.85	1.02	<0.01
		Caries risk and activity test	2.90	1.13	<0.01
		Risk assessment of periodontal disease	2.53	1.42	<0.01
		Malodor test	1.45	1.86	<0.01
		Examination of occlusal contact	1.39	0.95	<0.01
		Examination of dentition	1.39	0.66	<0.01
		Masticatory function test	1.92	1.28	<0.01
		Swallowing function test	1.37	1.30	<0.01
		Photo taking of oral cavity	1.57	-0.61	<0.01
	Periodontal treatment	Periodontal probing	3.07	-1.94	<0.01
		Scaling and root plaining	14.81	-1.91	<0.01
		SPT	4.94	-1.37	<0.01
		Assistance in periodontal surgery	1.53	-0.65	<0.01
	Oral function	Examination for patients with hypo oral function	17.78	0.46	<0.01
		Instruction for improvement oral function	14.97	0.40	<0.01
	Restorative procedures	Impressions for study casts	1.11	-1.96	<0.01
		Gingival retraction for tooth preparation	1.13	0.03	<0.01
		Impressions for inlay restoration	7.87	-0.77	<0.01
		Bite registration for inlay restoration	3.32	-0.79	<0.01
		Impressions for occlusal splints	18.73	-0.28	0.34
		Bite registration for occlusal splints	26.94	0.27	0.14
		Impressions for crown and bridge restoration	11.57	-0.69	<0.01
		Bite registration for crown and bridge restoration	4.01	-0.77	<0.01
		Impression by CAD/CAM	2.01	-0.21	<0.01
	Orthodontic treatment	Cephalometric Tracing	3.71	1.53	<0.01
		Banding	4.97	1.69	<0.01
		Bracket bonding	6.13	1.31	<0.01
		Impressions for parallel models for orthodontic treatments	20.91	0.92	<0.01
		Polishing and adjustment of orthodontic appliances	6.27	1.32	<0.01
		Removing orthodontic appliances	21.76	1.09	<0.01
		MFT	3.21	1.48	<0.01
	Dental implant	Probing around implants	11.73	0.31	<0.01
		Mobility test of the implant	7.82	0.30	<0.01
		Scaling around the implant	22.57	0.30	0.01
		Assistance in implant surgery	15.28	0.33	0.02
		Explanations after surgical treatment	14.82	0.34	<0.01
	Medical treatment	Infusion control	4.68	2.15	<0.01
		Venous access	7.43	2.17	<0.01
		Blood sampling	4.56	2.23	<0.01
		Infusion	6.93	2.12	<0.01
		Drug administration	5.69	2.01	<0.01
	Special care dentistry	Monitoring during dental treatment	2.52	1.62	<0.01
		Rubber dam isolation	1.67	1.94	<0.01

		Indirect training on swallowing dysfunction	6.18	1.55	<0.01
		Direct training on swallowing dysfunction	10.02	1.72	<0.01
		MFT	3.15	1.85	<0.01
		Tracheal aspiration during feeding training	4.90	2.26	<0.01
		Pharyngeal sputum	3.77	1.91	<0.01
		Behavior control	2.76	1.73	<0.01
		Oral hygiene management	4.12	0.74	<0.01
	Medical and dental instruction	Oral hygiene instructions for pediatric patients	2.79	-1.04	<0.01
		Oral hygiene instructions for patients with periodontal disease	29.67	-1.08	<0.01
		Instructions for handling dentures	10.40	-1.19	<0.01
		Instructions for fluoride mouthwash	1.88	-0.33	<0.01
		Smoking cessation guidance	1.76	0.43	<0.01
		Oral care for hospitalized patients	1.24	0.68	<0.01
		Consultations by telephone	1.33	0.62	<0.01
Dental office management		Reception work	4.16	-0.11	0.17
		Accounting of insurance	7.25	0.49	0.03
		Inputting data in a computer	4.47	0.17	0.21
		Sterilization and disinfection of dental equipment	3.35	-1.83	0.01
		Management and ordering of drugs and dental equipment	2.53	-1.15	<0.01
		Management and teaching for staff	1.10	-0.63	<0.01
		Training and management of dental hygienist students	0.45	1.94	0.01

Table S4. Mean and median of the ability calculated by IRT analysis of each group.

			Group 1 n=156	Group 2 n=264	Group 3 n=299	Group 4 n=1381	Group 5 n=529	Group 6 n=449	Group 7 n=428	Group 8 n=55	Group 9 n=235
Preventive dentistry		Mean +/- SD	0.03+/-0.48	0.15+/-0.35	-0.14+/-0.66	0.28+/-0.11	-0.18+/-0.75	-0.45+/-0.63	-0.76+/-0.77	-0.81+/-0.52	0.03+/-0.48
		Median(25th-75th)	0.29(0.29-0.29)	0.29(0.29-0.29)	0.29(-0.71-0.29)	0.29(0.29-0.29)	0.29(-0.71-0.29)	-0.71(-0.71-0.29)	-0.71(-1.35-0.29)	-0.71(-0.81--0.71)	0.29(0.29-0.29)
Assistant work	Medical Interview	Mean +/- SD	0.01+/-0.15	-0.17+/-0.5	-0.07+/-0.31	-0.71+/-0.78	-0.05+/-0.26	-0.18+/-0.46	-0.04+/-0.28	-0.01+/-0.21	0+/-0.18
		Median(25th-75th)	0.03(0.03-0.03)	0.03(0.03-0.03)	0.03(0.03-0.03)	-0.62(-1.52-0.03)	0.03(0.03-0.03)	0.03(0.03-0.03)	0.03(0.03-0.03)	0.03(0.03-0.03)	0.03(0.03-0.03)
	Examination	Mean +/- SD	0.61+/-0.72	0.11+/-0.81	0.06+/-0.78	-0.60+/-0.60	0.10+/-0.73	0.33+/-0.61	-0.50+/-0.63	0.70+/-0.68	0.40+/-0.69
		Median(25th-75th)	0.63(0.12-1.11)	0.15(-0.51-0.7)	-0.1(-0.51-0.59)	-0.62(-1.11--0.14)	0.12(-0.51-0.59)	0.21(-0.1-0.87)	-0.57(-1.11--0.14)	0.61(0.12-1.2)	0.38(-0.1-0.86)
	Periodontal treatment	Mean +/- SD	0.1+/-0.42	-0.16+/-0.68	0.24+/-0.25	-0.81+/-0.59	0.22+/-0.27	-1.36+/-0.58	-0.86+/-0.52	0.17+/-0.37	0.09+/-0.43
		Median(25th-75th)	0.3(0.3-0.3)	0.3(-0.64-0.3)	0.3(0.3-0.3)	-0.64(-1.24--0.64)	0.3(0.3-0.3)	-1.41(-2.07--0.64)	-0.64(-1.41--0.64)	0.3(0.3-0.3)	0.3(0.3-0.3)
	Oral function	Mean +/- SD	0.43+/-0.4	0.37+/-0.4	0.18+/-0.32	0.07+/-0.21	0.24+/-0.36	0.15+/-0.31	0.11+/-0.26	0.31+/-0.39	0.19+/-0.34
		Median(25th-75th)	0.6(0-0.83)	0(0-0.83)	0(0-0)	0(0-0)	0(0-0.6)	0(0-0)	0(0-0)	0(0-0.83)	0(0-0.6)
	Restorative procedures	Mean +/- SD	0.34+/-0.5	-0.03+/-0.71	0.05+/-0.63	-0.36+/-0.7	0.02+/-0.67	-1.02+/-0.23	-0.29+/-0.65	0.28+/-0.61	0.21+/-0.56
		Median(25th-75th)	0.34(0.18-0.76)	0.21(-0.68-0.76)	0.31(-0.61-0.34)	-0.22(-1.08-0.32)	0.31(-0.62-0.76)	-1.08(-1.08--1.08)	-0.22(-0.95-0.31)	0.34(0.11-0.76)	0.32(-0.04-0.76)
	Orthodontic treatment	Mean +/- SD	1.41+/-0.41	0.01+/-0.22	-0.01+/-0.14	0+/-0.17	0.01+/-0.21	1.47+/-0.52	0+/-0.19	0.66+/-0.87	1.34+/-0.42
		Median(25th-75th)	1.38(1.19-1.68)	-0.03(-0.03--0.03)	-0.03(-0.03--0.03)	-0.03(-0.03--0.03)	-0.03(-0.03--0.03)	1.43(1.31-1.74)	-0.03(-0.03--0.03)	-0.03(-0.03-1.54)	1.33(0.99-1.5)
	Dental implant	Mean +/- SD	0.68+/-0.48	0.24+/-0.53	0.37+/-0.54	-0.01+/-0.37	0.44+/-0.54	-0.12+/-0.27	-0.03+/-0.36	0.80+/-0.46	0.64+/-0.48
		Median(25th-75th)	0.75(0.52-1.08)	-0.21(-0.21-0.74)	0.51(-0.21-1.08)	-0.21(-0.21--0.21)	0.6(-0.21-1.08)	-0.21(-0.21--0.21)	-0.21(-0.21--0.21)	1.08(0.67-1.08)	0.75(0.51-1.08)
	Surgical treatment	Mean +/- SD	0+/-0	0+/-0.08	0+/-0	0+/-0.1	0+/-0	0+/-0	0+/-0	2.12+/-0.36	0+/-0
		Median(25th-75th)	0(0-0)	0(0-0)	0(0-0)	0(0-0)	0(0-0)	0(0-0)	0(0-0)	2.08(1.91-2.3)	0(0-0)
	Special care dentistry	Mean +/- SD	0.15+/-0.39	0.07+/-0.32	-0.04+/-0.18	-0.06+/-0.14	-0.03+/-0.18	0.07+/-0.31	-0.05+/-0.13	0.20+/-0.46	-0.01+/-0.22
		Median(25th-75th)	-0.08(-0.08-0.65)	-0.08(-0.08--0.05)	-0.08(-0.08--0.08)	-0.08(-0.08--0.08)	-0.08(-0.08--0.08)	-0.08(-0.08--0.05)	-0.08(-0.08--0.08)	-0.05(-0.08-0.68)	-0.08(-0.08--0.08)
	Health instructions	Mean +/- SD	0.49+/-0.66	0.14+/-0.76	-0.05+/-0.63	-0.48+/-0.58	0.03+/-0.74	-0.95+/-0.27	-0.33+/-0.59	0.29+/-0.72	0.09+/-0.66
		Median(25th-75th)	0.7(-0.1-1.29)	0.19(-0.52-0.7)	-0.1(-0.52-0.36)	-0.52(-0.86--0.1)	-0.1(-0.52-0.66)	-0.95(-1.25--0.79)	-0.52(-0.69--0.1)	0.36(-0.26-0.7)	0.19(-0.26-0.51)
Dental office management		Mean +/- SD	0.21+/-0.71	0.27+/-0.67	-0.02+/-0.81	0.02+/-0.71	-0.29+/-0.75	0.10+/-0.67	0.13+/-0.63	0.01+/-0.75	0.08+/-0.70
		Median(25th-75th)	0.26(-0.37-0.91)	0.35(-0.12-0.91)	0.01(-0.44-0.76)	0.01(-0.44-0.53)	-0.14(-0.64-0.23)	0.06(-0.37-0.76)	0.01(-0.14-0.63)	0.01(-0.44-0.76)	0.01(-0.37-0.79)

Table S5. Employment status in each group

	Regular attendee		Part time job		Total
	n	%	n	%	
Group 1	101	65%	55	35%	156
Group 2	175	66%	89	34%	264
Group 3	201	67%	98	33%	299
Group 4 *	726	53%	655	47%	1381
Group 5 *	278	53%	251	47%	529
Group 6 *	195	43%	254	57%	449
Group 7 *	238	56%	190	44%	428
Group 8	43	78%	12	22%	55
Group 9 *	107	46%	128	54%	235

The distributions were statistically significant by $\chi 2$ test($P<0.001$).

*indicate proportion of regular attendee and part time job was statistically significant by log linear tests. In this case, proportion of part time job was higher in each group.
